# Whole genome phylogenies for multiple *Drosophila* species

**DOI:** 10.1186/1756-0500-5-670

**Published:** 2012-12-04

**Authors:** Arun Seetharam, Gary W Stuart

**Affiliations:** 1 Department of Biology, Indiana State University, Terre Haute, Indiana, 47809, USA

**Keywords:** Singular value decomposition, Phylogenomics, Comparative genomics, *Drosophila* phylogeny

## Abstract

**Background:**

Reconstructing the evolutionary history of organisms using traditional phylogenetic methods may suffer from inaccurate sequence alignment. An alternative approach, particularly effective when whole genome sequences are available, is to employ methods that don’t use explicit sequence alignments. We extend a novel phylogenetic method based on Singular Value Decomposition (SVD) to reconstruct the phylogeny of 12 sequenced *Drosophila* species. SVD analysis provides accurate comparisons for a high fraction of sequences within whole genomes without the prior identification of orthologs or homologous sites. With this method all protein sequences are converted to peptide frequency vectors within a matrix that is decomposed to provide simplified vector representations for each protein of the genome in a reduced dimensional space. These vectors are summed together to provide a vector representation for each species, and the angle between these vectors provides distance measures that are used to construct species trees.

**Results:**

An unfiltered whole genome analysis (193,622 predicted proteins) strongly supports the currently accepted phylogeny for 12 *Drosophila* species at higher dimensions except for the generally accepted but difficult to discern sister relationship between *D. erecta* and *D. yakuba*. Also, in accordance with previous studies, many sequences appear to support alternative phylogenies. In this case, we observed grouping of *D. erecta* with *D. sechellia* when approximately 55% to 95% of the proteins were removed using a filter based on projection values or by reducing resolution by using fewer dimensions. Similar results were obtained when just the *melanogaster* subgroup was analyzed.

**Conclusions:**

These results indicate that using our novel phylogenetic method, it is possible to consult and interpret all predicted protein sequences within multiple whole genomes to produce accurate phylogenetic estimations of relatedness between *Drosophila* species. Furthermore, protein filtering can be effectively applied to reduce incongruence in the dataset as well as to generate alternative phylogenies.

## Background

Methods that determine phylogenies based on a restricted number of genes can be negatively affected by horizontal gene transfers, incomplete lineage-sorting, introgression, and the unrecognized comparison of paralogous genes. The recent explosive increase in the number of completely sequenced genomes allows us to consider inferring gene and/or organismal relationships using complete sequence data. Several methods for generating phylogenies based on whole genome information have been explored, and many of these have been applied to re-examine the phylogeny of *Drosophila*. These include methods based primarily or exclusively on gene content [1], gene order [2], and detailed comparisons of operationally defined orthologs [3]. However, these methods often fail to provide detailed and unbiased comparisons of a high fraction of sequences and instead produce phylogenies based on greatly filtered, preselected datasets. We developed a phylogenetic method that provides accurate comparisons for a high fraction of sequences within whole genomes without the prior identification of orthologous or homologous sites [4]. Our approach allows a relatively comprehensive comparison of complete genome protein sequence, thereby taking into account a higher fraction of total sequence information and providing comprehensive definitions for the various species of interest. This method has been successfully applied to a number of diverse species including vertebrate mitochondrial genomes, plant viral genomes, and eukaryotic nuclear genomes [4-7].

Complete genome sequences for 10 additional species of *Drosophila* were added to the sequences already available for *D. melanogaster* and *D. pseudoobscura* in order to improve the precision and sensitivity of evolutionary inference regarding these organisms [8]. As a result, the currently accepted species phylogeny for these organisms has been further refined and resolved. However, these methods generally continue to utilize greatly filtered data sets primarily comprised of selected single copy orthologous sequences [9-14].

Many such studies have resulted in what is largely considered to be a fully resolved phylogeny for the 12 sequenced species of *Drosophila.* However, some doubts remain with respect to the placement of certain members of the melanogaster group: *D. erecta, D. yakuba* and *D. melanogaster*, placement of the Hawaiian species: *D. grimshawi,* and to some extent *virilisrepleta* group: *D. virilis* and *D. mojavenis*[15-19]. Among these, the placement of *D. erecta* and *D. yakuba* with respect to *D. melanogaster* is perhaps least certain. Though evidence has been presented to support all the possible phylogenies with respect to *D. melanogaster, D. erecta,* and *D. yakuba,* support for each of these phylogenies is not uniformly strong [12]. In this study we apply our more inclusive whole genome phylogenetic method on the 12 genomes of *Drosophila* to further investigate and validate our current understanding of their phylogenetic relationships.

## Results and discussion

Preliminary studies were conducted using a small dataset comprising only 6 genomes of the melanogaster group (*D. melanogaster, D. sechellia, D. simulans, D. erecta, D. yakuba and D. ananassae*) with a total of 100,851 predicted proteins. Further studies were conducted using a large dataset consisting of all the 12 *Drosophila* spp. genomes with a total of 193,622 proteins (Table 1). Additional 11 genome datasets excluding one of the *melanogaster* group species were also constructed for the detailed analysis of the phylogenies. Although there were large similarities in the total number of genes among the *Drosophila* species, there were large variations in the total number of predicted proteins (Table 1). It seems likely that the melanogaster genome is more fully annotated with a larger number of alternatively spliced transcripts producing multiple (but perhaps slightly different) protein products relative to other *Drosophila* genomes. Among the 12 species, *D. melanogaster* had the highest number of predicted proteins (22,765) and *D. virilis* had the lowest (14,491). Each species’ contribution to the dataset was in the range of 7.48% to 8.51% except for *D. melanogaster* which contributed about 11.76% for the total. In previous studies, we noted that a modest size difference in genomes has little effect on the final outcome of the tree [4,6].

**Table 1:** **List of 12 *Drosophila *spp used in the analysis, along with the number of predicted proteins**

***#***	*** Species***	***Genes***	***Proteins***
1	*Drosophila simulans*	16117	15415
2	*Drosophila sechellia*	17286	16471
3	*Drosophila melanaogaster*	15431	22765
4	*Drosophila erecta*	15810	15048
5	*Drosophila ananassae*	15978	15070
6	*Drosophila yakuba*	16904	16082
7	*Drosophila pseudoobscura*	16712	16308
8	*Drosophila persimilis*	17573	16878
9	*Drosophila willistoni*	16385	15513
10	*Drosophila mojavensis*	15179	14595
11	*Drosophila virilis*	15343	14491
12	*Drosophila grimshawi*	15885	14986

### Higher dimension SVD analysis

**Figure 1:**
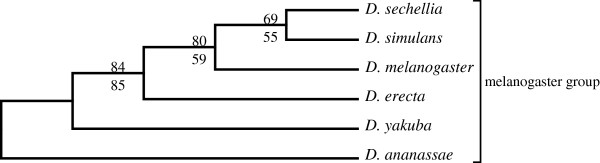
**The higher dimension SVD tree for the 6 *Drosophila *spp., using all 700 vectors, without filtering any proteins (upper branch values, modified jackknife and lower branch values, bootstrap procedure for tree generation).**

**Figure 2:**
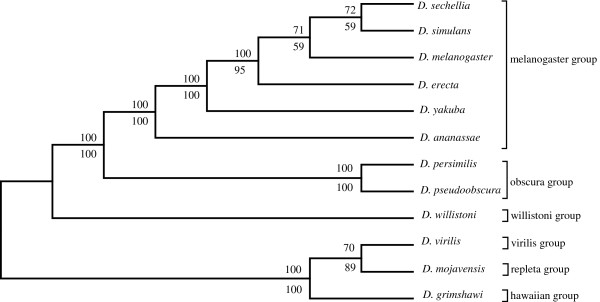
**The higher dimension SVD tree for the 12 *Drosophila *spp., using all 700 vectors, without filtering any proteins (upper branch values, modified jackknife and lower branch values, bootstrap procedure for tree generation).**

Figure 1 and 2 shows the SVD-based topology obtained via Neighbor-joining for the 6 and 12 genome *Drosophila* species data sets respectively. Two types of resampling methods were used to estimate branch statistics for this tree. The bottom value on each branch was generated using a traditional bootstrap procedure [4] by sampling 800 singular triplets to construct 700 species trees. The top value on each branch was generated using a successive, delete-one jackknife procedure [4] wherein the least dominant singular vector was removed successively (from 800 to 100 vectors) to generate 700 ordered sets of singular vectors, and a new tree was estimated following each removal. Most of the branches were well supported following application of either the modified jackknife procedure or the bootstrap procedure. Bootstrap yielded a slightly lower branch support for the *D. sechellia*, *D. simulans,* and *D. melanogaster* branch but all other branches were strongly supported by both procedures. The observed difference was likely due to the uniform use of the 700 most dominant vectors in our modified jackknife procedure, while in contrast, the standard bootstrap samples randomly over all 800 vectors generated. The end result is a phylogeny that corresponds well to the currently accepted phylogeny [12,20-22], except for *D. erecta* and *D. yakuba*, which remain adjacent in the tree, but fail to cluster as sister species.

In order to further examine the robustness of the data supporting the correct tree, we performed a series of analyses by systematically excluding protein sequences that were poorly described by their corresponding singular vectors in terms of projection values. The theoretical projection values for a given protein range from −1 to +1. In the first step, all protein sequences having projection value less than or equal +0.001 and more than or equal to −0.001 were removed (about 9,500 sequences). The filter was increased stepwise with an increment of 0.001 and each corresponding dataset was used in turn to construct a tree. When about 54.54% (105,596 sequences) of the original dataset was removed (projection value less than or equal to +0.003 and more than or equal to −0.003), a unique clustering of *D. erecta* with *D. schelliea* was observed (Additional file 1). Continued successive increases in stringency to remove poorly described proteins failed to alter this novel cluster until more than 95% (185,039) of the total protein sequences were removed. This resulted in a re-clustering of *D. erecta* with *D. yakuba* as sister species, but this was accompanied by the movement of *D. melanogaster* to a novel position (Additional file 2). Removing a high fraction of poorly described proteins (those with smaller projections on any singular vector) would presumably tend to produce a more highly correlated data set consisting of smaller sets of highly conserved proteins. The tree generated using the modified jack knife procedure, rather than the bootstrap, showed a similar branching pattern. Branch support values for the tree exceeded 80% in all cases, and only 60% for the *D. yakuba and D. erecta* cluster.

### Lower dimension SVD analysis

**Figure 3:**
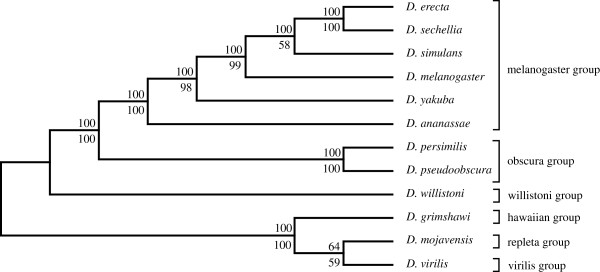
**The lower dimension SVD tree for the 12 *Drosophila *spp., using 300 vectors, without filtering any proteins (upper branch values, modified jackknife and lower branch values, bootstrap procedure for tree generation).**

**Figure 4:**
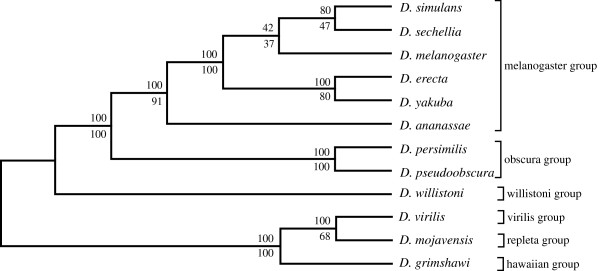
**The lower dimension SVD tree for the 12 *Drosophila *spp., using 300 vectors, with heavy filtering of proteins with projection values ≤ ±0.035.** A total of 4430 (2.43%) proteins were used for constructing trees (upper branch values, modified jackknife and lower branch values, bootstrap procedure for tree generation).

A corresponding lower dimension analyses of the *Drosophila* spp. was also conducted using the same procedure but with fewer (500) singular triplets. Here the bootstrap branch statistics were generated by sampling 100 random sets of 150 singular triplets to construct 100 species trees. The delete-one jackknife values were generated using 400 ordered sets of singular vectors. Trees were estimated following each successive removal of a least dominant vector from 500 to 100 vectors. The SVD phylogeny obtained for the unfiltered 12 *Drosophila* species dataset (Figure 3) corresponds well to the currently accepted phylogeny, except for *D. erecta*, which shows a novel affinity with *D. sechellia*. It proved possible to disrupt this novel affinity after reducing the number of proteins used in the summation step by 97.57% (Figure 4) by applying a relatively severe filter (projection value less than or equal to +0.035 and more than or equal to −0.035) and thus using only the remaining highly correlated data set consisting of smaller sets of highly conserved proteins. Branch support values for the tree exceeded 70% in all cases, and more than 80% except for the *D. melanogaster, D. yakuba and D. erecta* cluster.

**Figure 5:**
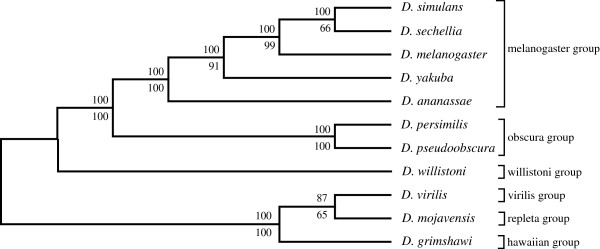
**The lower dimension SVD tree for the 11 *Drosophila *species (excluding *D. erecta*) using 300 vectors, without filtering any proteins (upper branch values, modified jackknife and lower branch values, bootstrap procedure for tree generation).**

In order to study the relationships among members of the *melanogaster* group without the influence of *D. erecta*, a slightly smaller dataset of 11 *Drosophila* species (178,574 total predicted proteins) was used for analysis. This data set produced the currently accepted phylogeny with strong branch support (Figure 5) [12,20-22]. The observed relationship was consistent across different levels of protein filtering. Both the bootstrap and the modified jackknife produced strong branch support values for most branches.

**Figure 6:**
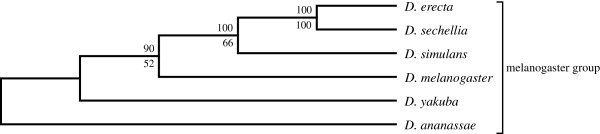
**The lower dimension SVD tree for the 6 *Drosophila *species (melanogaster group) using 300 vectors, without filtering any proteins (upper branch values, modified jackknife and lower branch values, bootstrap procedure for tree generation).**

**Figure 7:**
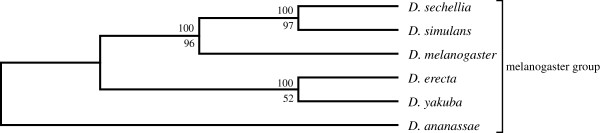
**The lower dimension SVD tree for the 6 *Drosophila *spp., using 300 vectors, with heavy filtering of proteins with projection values ≤ ±0.035.** A total of 4048 (4.06%) proteins were used for constructing trees (upper branch values, modified jackknife and lower branch values, bootstrap procedure for tree generation).

**Figure 8:**
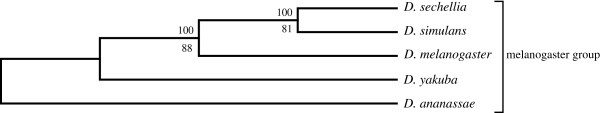
**The lower dimension SVD tree for the 5 *Drosophila *species (melanogaster group, excluding *D. erecta*) using 300 vectors, without filtering any proteins (upper branch values, modified jackknife and lower branch values, bootstrap procedure for tree generation).**

A similar result was obtained with an even smaller dataset that included only 6 genomes with 100,851 predicted proteins (Figure 6 and 7). When subjected to SVD analysis, this produced the currently accepted phylogeny for all 6 members of the *melanogaster* group, but only under stringent protein filtering (Figure 7). The effect of including more proteins using a less severe protein filter was similar for both the 12 genome tree and the 6 genome tree: *D. erecta* fails to cluster with *D. yakuba* and instead clusters with *D. sechellia*. However, just like in the 11 *Drosophila* dataset, exclusion of *D. erecta* from the melanogaster group produced the currently accepted phylogeny with strong branch support (Figure 8) without filtering any proteins. The effect of other genomes on the phylogeny was systematically studied by excluding one of the *melanogaster* group species from the original 12 genome dataset. All these analyses showed the novel *D. sechellia* and *D. erecta* clustering (Additional file 3, Additional file 4, Additional file 5 and Additional file 6) except for the dataset from which *D. sechellia* was excluded which produced the currently accepted phylogeny (Additional file 7). But, all datasets produced the currently accepted phylogeny under stringent filtering conditions (Additional files 8, 9, 10, 11, and 12).

## Conclusions

Our results indicate that it is possible to consult and interpret all predicted protein sequences within multiple whole genomes to produce accurate phylogenetic estimations of relatedness between *Drosophila* species. Unlike our approach, the most recent independent standard analyses based on whole genome sequence information depend upon filtered data sets in which a restricted number of highly conserved and putatively orthologous genes were compared. In addition, unlike standard methods which use sequence alignments, our method uses angles between high dimensional vectors to estimate evolutionary distance. Despite these novelties in method, the phylogenetic tree derived for the 6 species of the melanogaster group, as well as all 12 species of *Drosophila,* exhibits strong branch support values and corresponds almost exactly to the currently accepted phylogeny. We conclude that it is possible to include the entire dataset for a more inclusive and potentially more robust analysis using a novel method to produce equivalent results.

This greatly expanded data set appears to contain a strong component of conflicting sequence information that specifically causes *D. erecta* and *D. sechellia* to cluster, but this was observed only when more than 55% (105,596) of the proteins are removed. However, this cluster disappears again when 95% (185,039) of poorly described proteins are removed. At lower dimensions, the *D. erecta* and *D. sechellia* cluster appears to be stable under various filter settings. Only under stringent filtering conditions could the correct phylogeny be restored. Additionally exclusion of either *D. sechellia* or *D. erecta* from the 12 species dataset could produce the currently accepted phylogeny.

The relative placement of *D. erecta* and *D. yakuba* with respect to *D. melanogaster* was largely uncertain until multigene analyses tended to support the same standard tree [9-14,23]. This standard tree is well supported in multiple distinct analyses and is essentially non-controversial, representing the currently accepted statement concerning the relatedness of the first twelve fully sequenced *Drosophila* genomes. However, previous single gene analyses supported a variety of distinct trees [15,16,24-30], and more comprehensive surveys of putative orthologs revealed a high frequency of conflicting trees [11-13]. Even though the currently accepted phylogeny had the strongest support, depending on the evolutionary model applied, roughly 40% of all orthologous genes examined supported alternative phylogenies within the *melanogaster* subgroup [12]. In this case, the standard *D. erecta/D. yakuba* cluster was specifically examined, and only two alternatives, those in which either of these species specifically clustered instead with *D. melanogaster*, were considered. Two reasons are commonly offered to explain the conflicts observed in these surveys of single gene phylogenies: incomplete lineage sorting, and introgression. Either of these processes could potentially be at least partly responsible for the novel grouping of *D. erecta* and *D. sechellia* we observed under the special mid-range filtering conditions reported here.

An alternative but not mutually exclusive explanation for the conditional novel clustering observed in this work is that the sequence signal causing this exists primarily outside of a reasonably complete list of identifiable orthologs (Additional file 2). Although not a necessity, this signal could easily be interpreted as homoplasious. This interpretation is supported by the fact that the standard clustering of *D. yakuba* and *D. erecta* was observed again when using only protein sequences with the highest projection values, which includes a small subset of proteins that are more likely to represent close homologs or orthologs. It is also possible that the sequence signal responsible might not be exclusively located outside identifiable orthologs, but might also be partly embedded within orthologs as similar subsets of specific sequence changes within these genes. In either case, it would still be interesting to further investigate the source and strength of these presumed homoplasies, given that they specifically and consistently support a single alternative placement for a single species within a complex tree.

Regardless of their location relative to orthologs, if the sequence characteristics within our all-inclusive analysis that consistently result in the association of *D.erecta* with *D. sechellia* represent homoplasious molecular responses to one or more environmental conditions, then this represents a third widely recognized mechanism for generating phylogenetic conflict within sequence data: adaptive convergence. Hence the affinity observed here between *erecta* and *sechellia* could result from non-random homoplasy with evolutionary significance. As an example for illustration, consider that *D. sechellia* and *D. erecta* are two of only three “specialist” species in the phylogeny that have adapted to specific food sources, and unlike the third species (*virilis*), they are closely related members of the *melanogaster* subgroup and have both adapted to particular fruits [31]. Although this single proposed adaptation might seem unlikely to be the sole source of a homoplasious signal capable of clustering *D. sechillia* and *D. erecta* in our analysis, multiple similar undiscovered or undescribed convergences could produce a sufficiently robust signal.

## Methods

### Datasets

Complete predicted protein sequences for 12 *Drosophila* species were downloaded from the ‘Assembly, Alignment and Annotation of 12 *Drosophila* species’ website (http://rana.lbl.gov/drosophila/) and were compiled into a single dataset. Various distinct subsets of this larger dataset were also constructed. The number of protein sequences found within the genome of each species of *Drosophila* is summarized in Table 1.

### Peptide frequencies and SVD

The twenty amino acids provide 160,000 possible tetrapeptides, defining each row of the peptide frequency matrix. For every protein, the frequency of each of these tetrapeptides formed the columns of the matrix. The resulting matrix is thus a peptide frequency matrix *(A)*, with each column providing protein vector definitions using 160,000 separate tetrapeptide frequency elements. In our previous studies, using tripeptides we were able to estimate similarities between highly divergent, small set of proteins [32]. It was also shown that tetrapeptides work better for larger data sets derived from vertebrate mitochondrial genomes or whole bacterial genomes [4]. Since, pentapeptides did not add any resolution for estimating similarities on our simulated datasets (unpublished); we chose tetrapeptides for constructing frequency matrix. A peptide frequency matrix was generated for all the three datasets, separately. The resulting matrix was then subjected to a truncated SVD analysis that generates three component matrices: the “left” matrix or “peptide” matrix (U), the “right” matrix or “protein” matrix (V) and the central matrix (∑). The original matrix can be reformed using the relation *A = U* ∑ *V*^*T*^. The “protein” vectors provided in the “right” factor matrix are known to provide reduced dimensional definitions for all proteins in the dataset as linear combinations of the orthogonal “right” singular vectors [6]. The dataset could produce a total of 910 singular vectors with the reduced dimensional space. An examination of the contribution provided by the less dominant singular vectors showed that these vectors tended to decrease the resolution of the resulting phylogenetic tree (not shown). Using the first 800 vectors was thus determined to be sufficient. The current phylogenetic studies were conducted under two different SVD settings, one referred as “higher dimension,” where we used a total of 800 singular triplets as output and the other referred as “lower dimension” using only 400 singular triplets as output. The SVD was then applied to the 12, 11 and 6 species datasets of *Drosophila* separately. Three output matrices were obtained consisting of 800 (for higher dimension analysis) and 500 (for lower dimension analysis) singular triplets (left and right singular vectors and their corresponding singular value). Higher the value of vector elements, most dominant is the singular vector and these singular vectors define one or two conserved gene families (or subfamilies) as particular linear combinations of proteins. The detailed comparative information contained within the hundreds of singular vectors and their corresponding motifs and gene families was subsequently used to build a species phylogeny by summing all the SVD-derived right protein vectors separately for each organism and then comparing the relative orientation of the resulting species vectors [6].

### Filtering proteins

A systematic exclusion of protein sequences, based on their projection values were done to filter poorly described proteins. The projection value represent a given protein range from −1 to +1. In the first step, all protein sequences having projection value less than or equal +0.001 and more than or equal to −0.001 were removed (about 9,500 sequences). The filter was increased stepwise with an increment of 0.001 and each corresponding dataset was used in turn to construct a tree.

### Species trees and branch support

Distance matrices were derived by summing all the SVD derived right protein vectors for a given organism and then comparing the relative orientation of the resulting species vectors using the program cosdist. Species trees were subsequently derived from distance matrices using Phylip-Neighbor. Two distinct resampling methods were used to provide branch support: a traditional bootstrap procedure and a modified jackknife procedure. For the bootstrap, a fixed number of singular vectors were randomly sampled from the total singular vectors generated and were used to construct 100 species trees. For the successive delete-one jackknife procedure [4-7], the least dominant singular vector was removed successively (from the total vectors generated, down to 100 vectors) to generate ordered sets of singular vectors, and a new tree was estimated following each removal.

## Competing interests

The authors declare that there are no competing interests.

## Authors’ contributions

GS established the overall concept and approach, and AS completed the phylogenetic analysis, as well as producing all tables, figures, and writing early drafts of the manuscript. All authors read and approved the final manuscript.

## Supplementary Material

Additional file 1 **SVD (higher dimension) tree for the 12 *Drosophila *spp., using all 700 vectors, with filtering cut off value of ±0.003, retaining 88,026 (45.46%) protein sequences (upper branch values, modified jackknife and lower branch values, bootstrap procedure for tree generation).** Click here for file

Additional file 2 **SVD (higher dimension) tree for the 12 *Drosophila *spp., using all 700 vectors, with filtering cut off value of ±0.032, retaining 8,583 (4.43%) protein sequences (upper branch values, modified jackknife and lower branch values, bootstrap procedure for tree generation).** Click here for file

Additional file 3 **SVD (lower dimension) tree for the 11 *Drosophila *species (excluding *D. melanogaster*), using 300 vectors, without filtering any proteins (upper branch values, modified jackknife and lower branch values, bootstrap procedure for tree generation).** Click here for file

Additional file 4 **SVD (lower dimension) tree for the 11 *Drosophila *species (excluding *D. simulans *using 300 vectors, without filtering any proteins (upper branch values, modified jackknife and lower branch values, bootstrap procedure for tree generation).** Click here for file

Additional file 5 **SVD (lower dimension) tree for the 11 *Drosophila *species (excluding *D. ananassae*) using 300 vectors, without filtering any proteins (upper branch values, modified jackknife and lower branch values, bootstrap procedure for tree generation).** Click here for file

Additional file 6 **SVD (lower dimension) tree for the 11 *Drosophila *species (excluding *D. yakuba*) using 300 vectors, without filtering any proteins (upper branch values, modified jackknife and lower branch values, bootstrap procedure for tree generation).** Click here for file

Additional file 7 **SVD (lower dimension) tree for the 11*Drosophila *species (excluding *D. sechellia*) using 300 vectors, without filtering any proteins (upper branch values, modified jackknife and lower branch values, bootstrap procedure for tree generation).** Click here for file

Additional file 8 **SVD (lower dimension) tree for the 11*Drosophila *species (excluding *D. melanogaster*), using 300 vectors, with filtering cut off value of ±0.035, retaining 4146 (2.43%) protein sequences (upper branch values, modified jackknife and lower branch values, bootstrap procedure for tree generation).** Click here for file

Additional file 9 **SVD (lower dimension) tree for the 11 *Drosophila *species (excluding *D. sechellia*), using 300 vectors, with filtering cut off value of ±0.035, retaining 4271 (2.43%) protein sequences (upper branch values, modified jackknife and lower branch values, bootstrap procedure for tree generation).** Click here for file

Additional file 10 **SVD (lower dimension) tree for the 11 *Drosophila *species (excluding *D. simulans*), using 300 vectors, with filtering cut off value of ±0.035, retaining 4611 (2.61%) protein sequences (upper branch values, modified jackknife and lower branch values, bootstrap procedure for tree generation).** Click here for file

Additional file 11 **SVD (lower dimension) tree for the 11 *Drosophila *species (excluding *D. ananassae*), using 300 vectors, with filtering cut off value of ±0.035, retaining 4343 (2.45%) protein sequences (upper branch values, modified jackknife and lower branch values, bootstrap procedure for tree generation).** Click here for file

Additional file 12 **SVD (lower dimension) tree for the 11 *Drosophila *species (excluding *D. yakuba*), using 300 vectors, with filtering cut off value of ±0.035, retaining of 4628 (2.63%) protein sequences (upper branch values, modified jackknife and lower branch values, bootstrap procedure for tree generation).** Click here for file
