# Supplementary figures and images for: Whole genome phylogenies for multiple Drosophila species
Source: BMC Res Notes. 2012 Dec 4;5:670. doi: 10.1186/1756-0500-5-670 (PMC3531268; doi:10.1186/1756-0500-5-670)

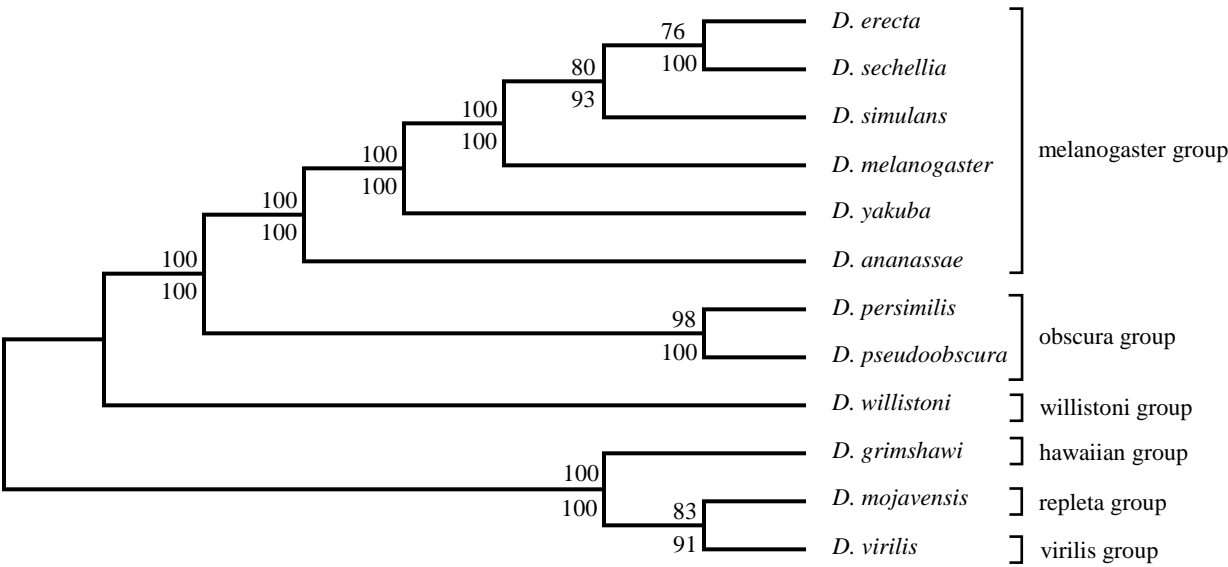

Supplement: Additional file 1 — SVD (higher dimension) tree for the 12 Drosophila spp., using all 700 vectors, with filtering cut off value of ±0.003, retaining 88,026 (45.46%) protein sequences (upper branch values, modified jackknife and lower branch values, bootstrap procedure for tree generation). [file 1756-0500-5-670-S1.pdf]

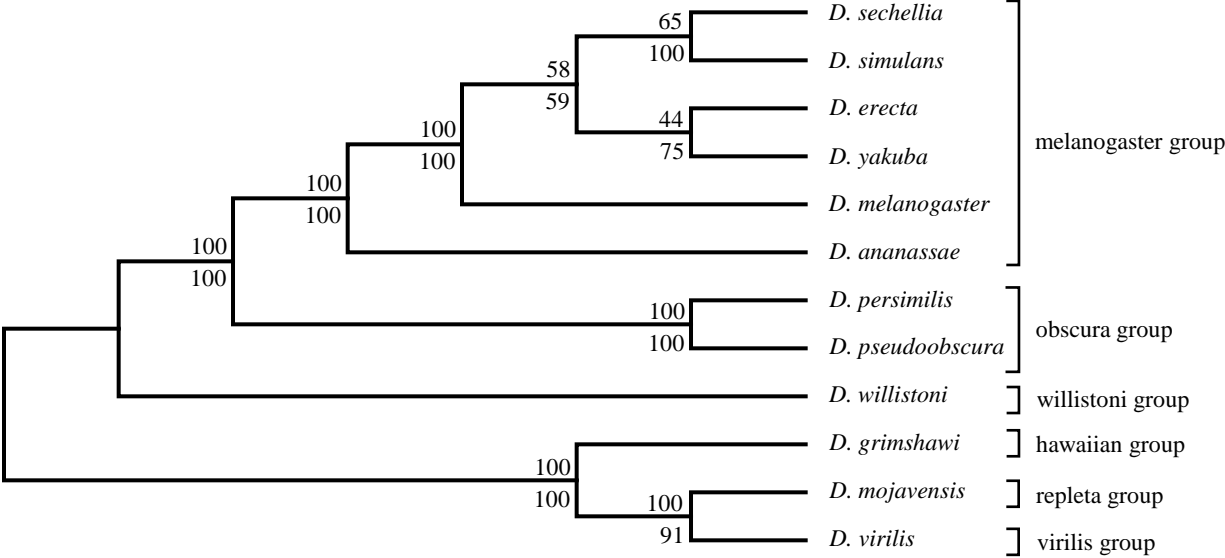

Supplement: Additional file 2 — SVD (higher dimension) tree for the 12 Drosophila spp., using all 700 vectors, with filtering cut off value of ±0.032, retaining 8,583 (4.43%) protein sequences (upper branch values, modified jackknife and lower branch values, bootstrap procedure for tree generation). [file 1756-0500-5-670-S2.pdf]

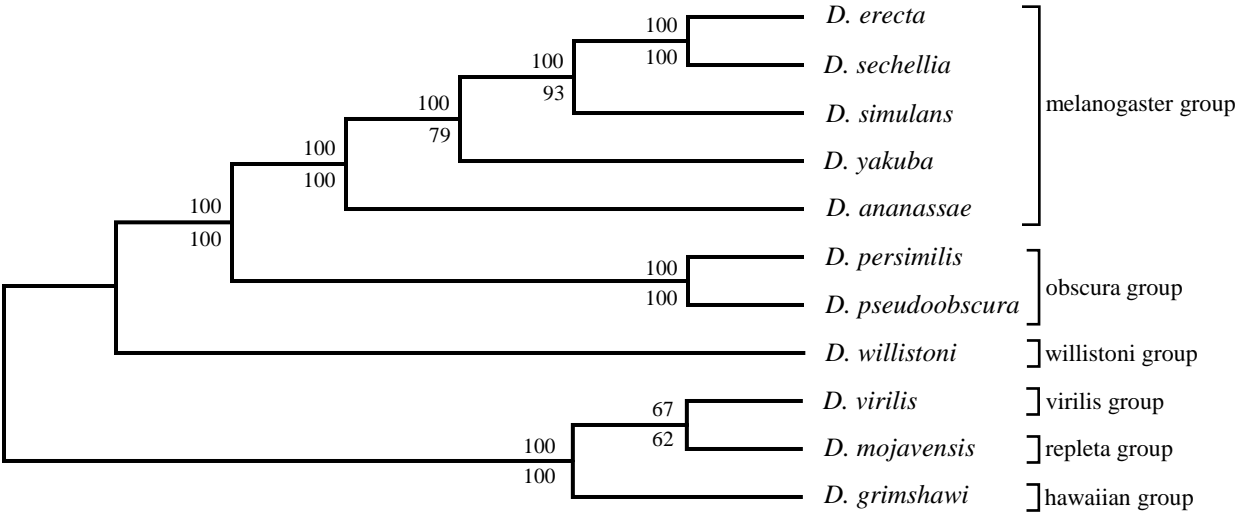

Supplement: Additional file 3 — SVD (lower dimension) tree for the 11 Drosophila species (excluding D. melanogaster), using 300 vectors, without filtering any proteins (upper branch values, modified jackknife and lower branch values, bootstrap procedure for tree generation). [file 1756-0500-5-670-S3.pdf]

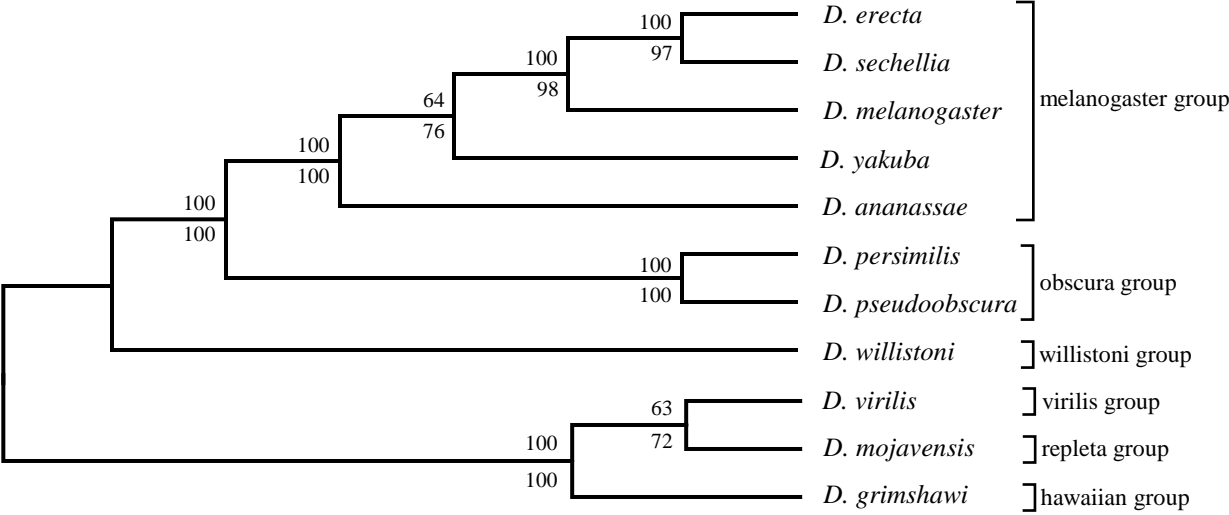

Supplement: Additional file 4 — SVD (lower dimension) tree for the 11 Drosophila species (excluding D. simulans using 300 vectors, without filtering any proteins (upper branch values, modified jackknife and lower branch values, bootstrap procedure for tree generation). [file 1756-0500-5-670-S4.pdf]

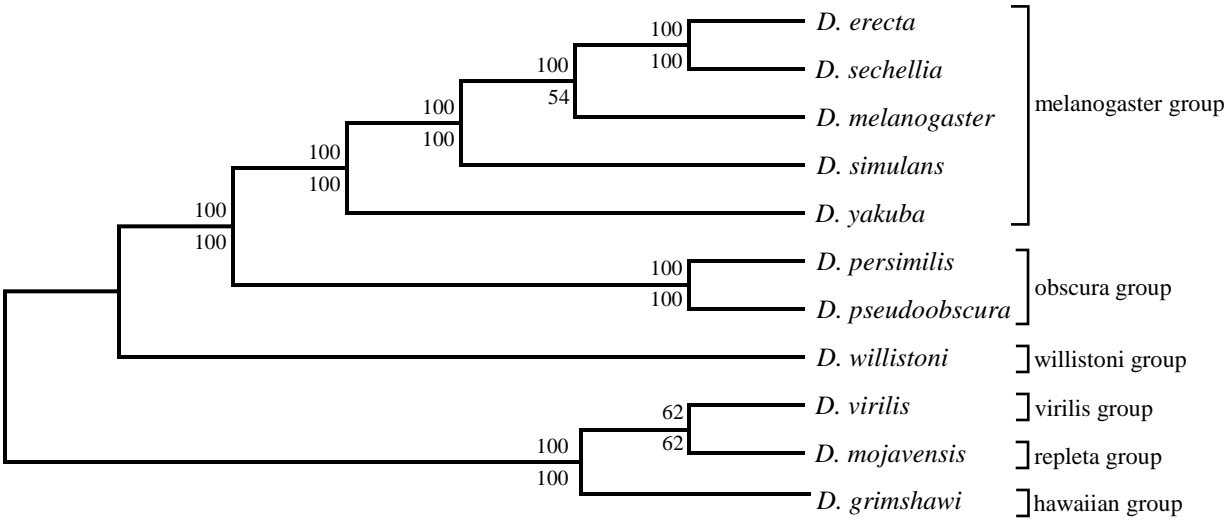

Supplement: Additional file 5 — SVD (lower dimension) tree for the 11 Drosophila species (excluding D. ananassae) using 300 vectors, without filtering any proteins (upper branch values, modified jackknife and lower branch values, bootstrap procedure for tree generation). [file 1756-0500-5-670-S5.pdf]

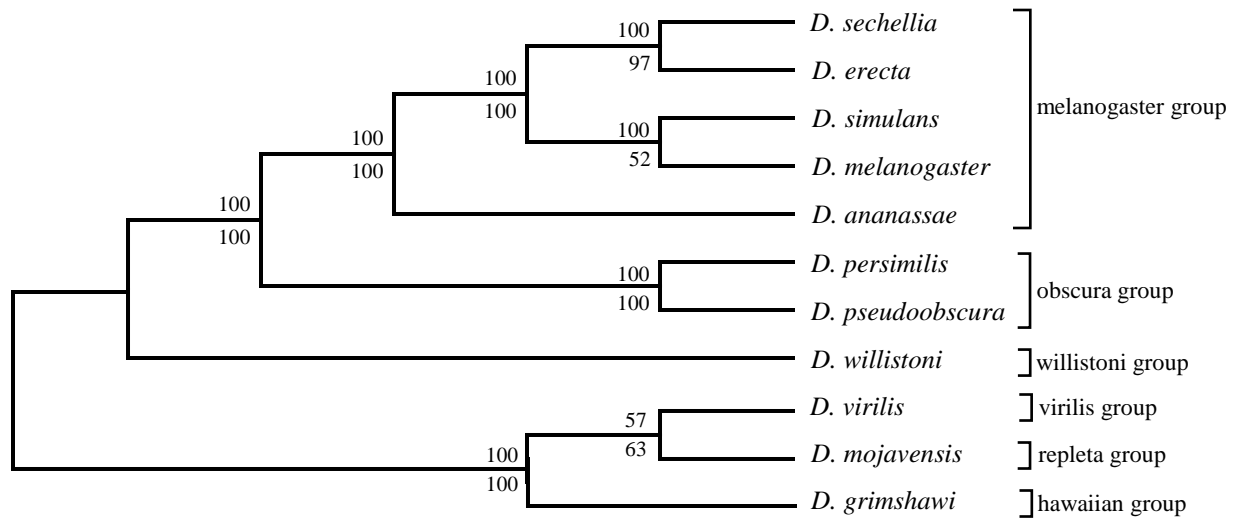

Supplement: Additional file 6 — SVD (lower dimension) tree for the 11 Drosophila species (excluding D. yakuba) using 300 vectors, without filtering any proteins (upper branch values, modified jackknife and lower branch values, bootstrap procedure for tree generation). [file 1756-0500-5-670-S6.pdf]

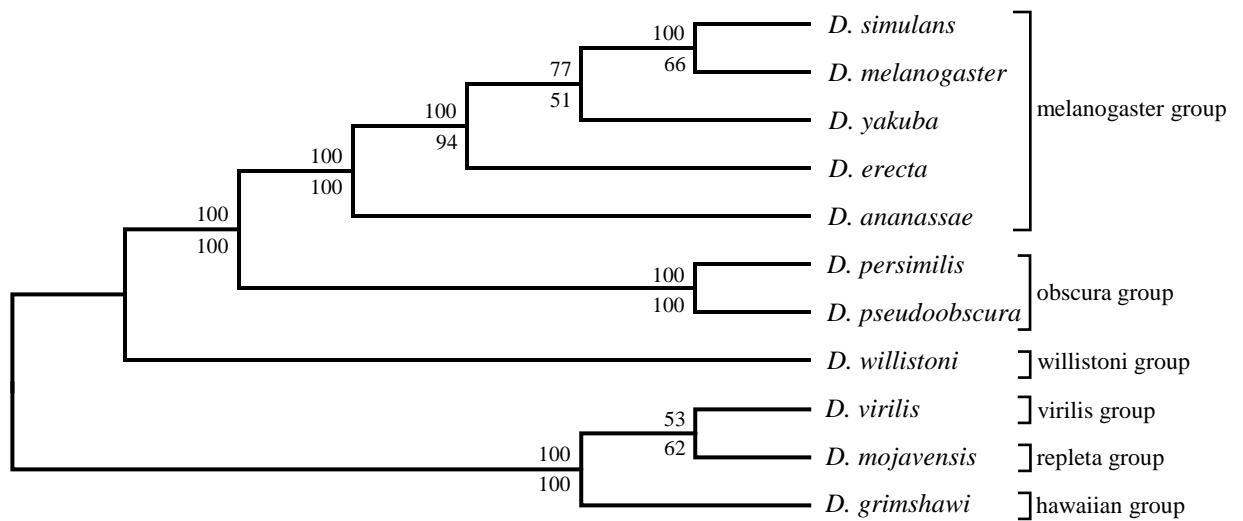

Supplement: Additional file 7 — SVD (lower dimension) tree for the 11Drosophila species (excluding D. sechellia) using 300 vectors, without filtering any proteins (upper branch values, modified jackknife and lower branch values, bootstrap procedure for tree generation). [file 1756-0500-5-670-S7.pdf]

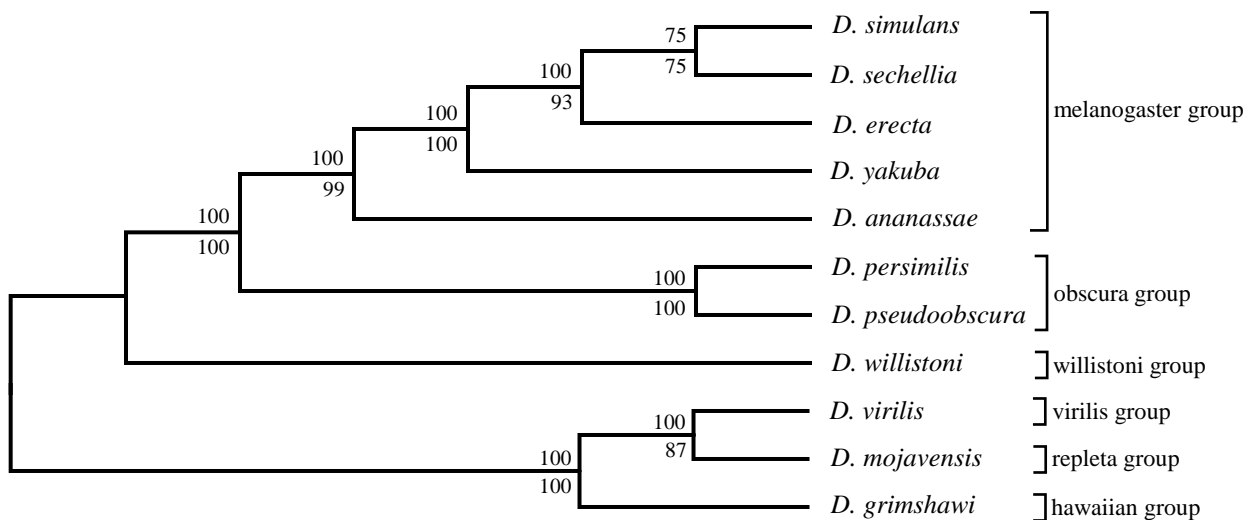

Supplement: Additional file 8 — SVD (lower dimension) tree for the 11Drosophila species (excluding D. melanogaster), using 300 vectors, with filtering cut off value of ±0.035, retaining 4146 (2.43%) protein sequences (upper branch values, modified jackknife and lower branch values, bootstrap procedure for tree generation). [file 1756-0500-5-670-S8.pdf]

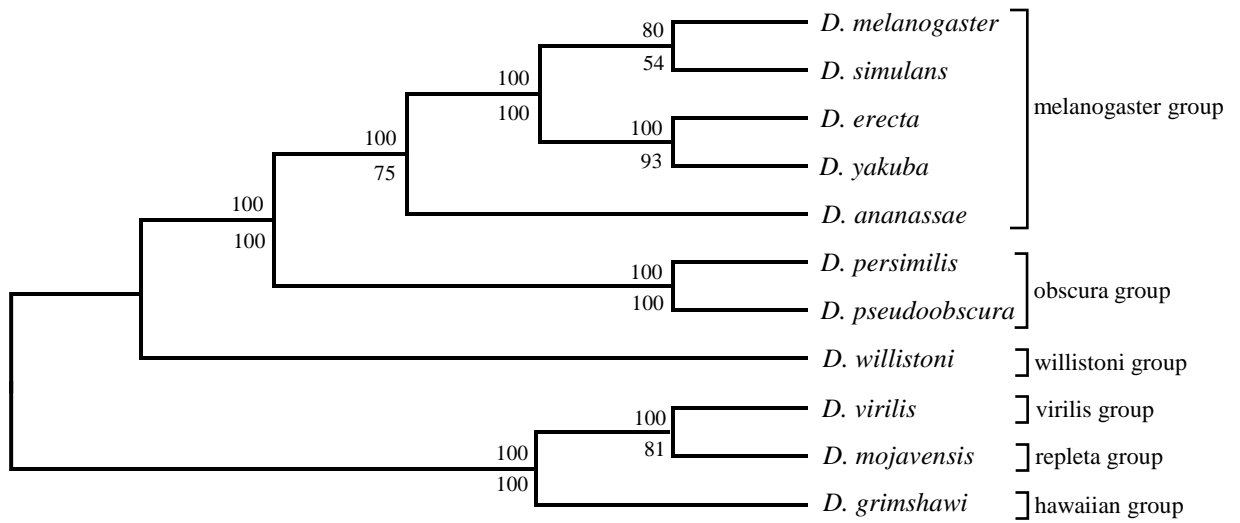

Supplement: Additional file 9 — SVD (lower dimension) tree for the 11 Drosophila species (excluding D. sechellia), using 300 vectors, with filtering cut off value of ±0.035, retaining 4271 (2.43%) protein sequences (upper branch values, modified jackknife and lower branch values, bootstrap procedure for tree generation). [file 1756-0500-5-670-S9.pdf]

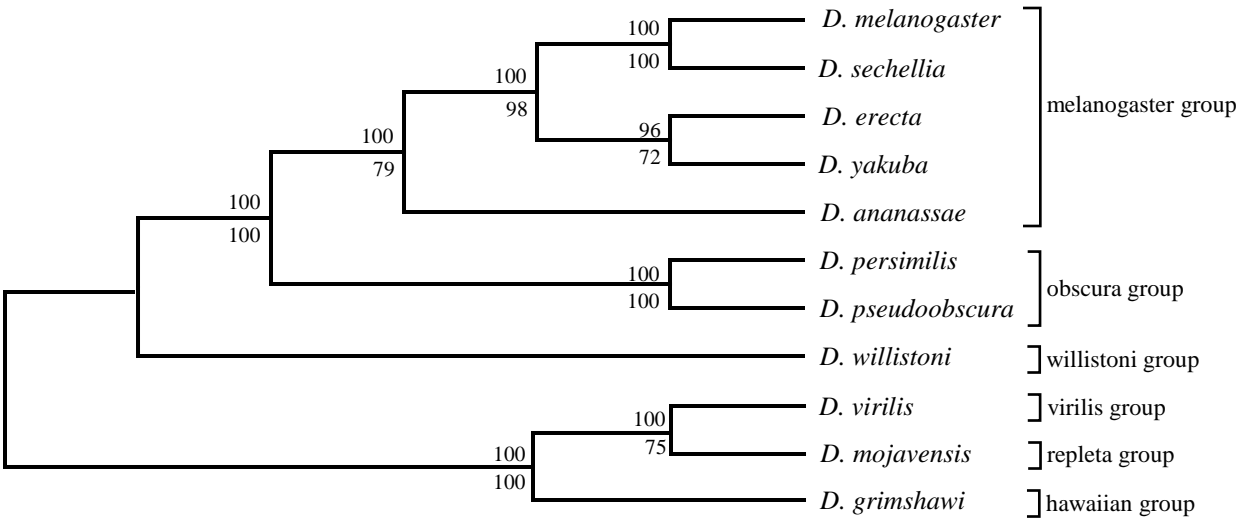

Supplement: Additional file 10 — SVD (lower dimension) tree for the 11 Drosophila species (excluding D. simulans), using 300 vectors, with filtering cut off value of ±0.035, retaining 4611 (2.61%) protein sequences (upper branch values, modified jackknife and lower branch values, bootstrap procedure for tree generation). [file 1756-0500-5-670-S10.pdf]

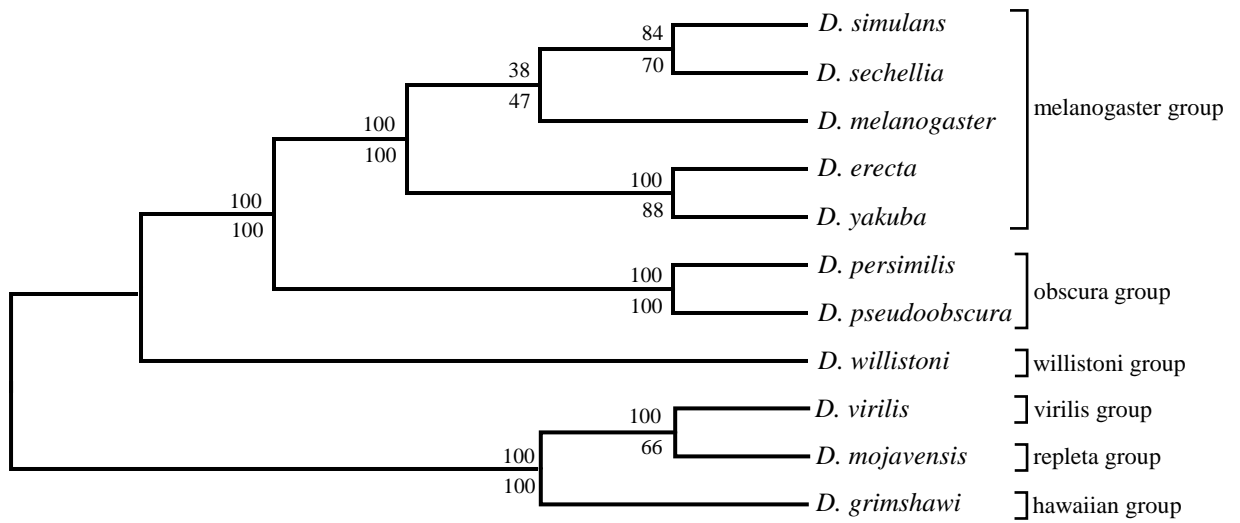

Supplement: Additional file 11 — SVD (lower dimension) tree for the 11 Drosophila species (excluding D. ananassae), using 300 vectors, with filtering cut off value of ±0.035, retaining 4343 (2.45%) protein sequences (upper branch values, modified jackknife and lower branch values, bootstrap procedure for tree generation). [file 1756-0500-5-670-S11.pdf]

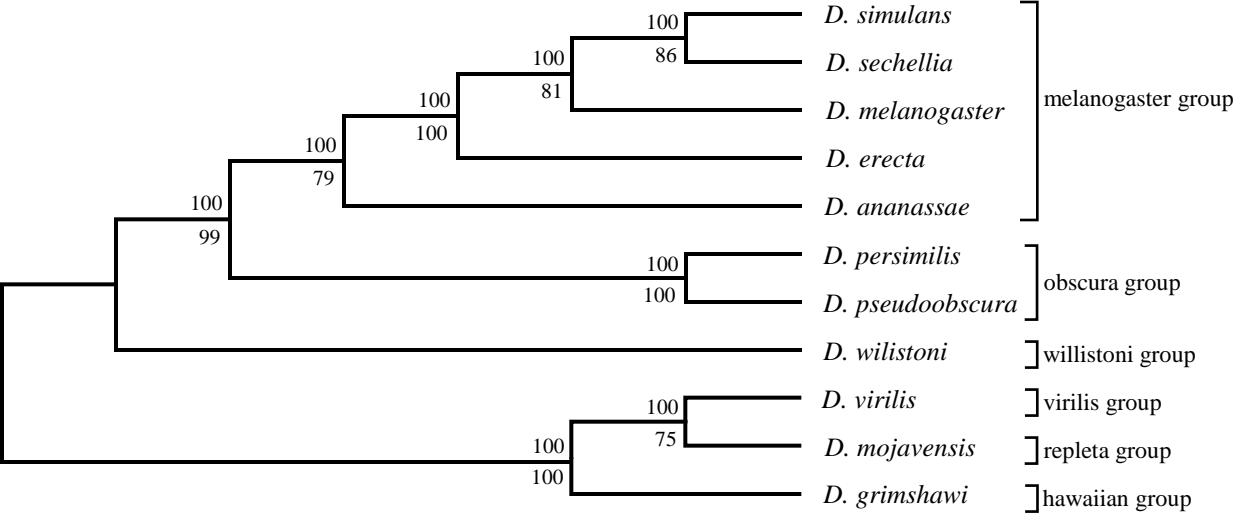

Supplement: Additional file 12 — SVD (lower dimension) tree for the 11 Drosophila species (excluding D. yakuba), using 300 vectors, with filtering cut off value of ±0.035, retaining of 4628 (2.63%) protein sequences (upper branch values, modified jackknife and lower branch values, bootstrap procedure for tree generation). [file 1756-0500-5-670-S12.pdf]
